# The Effects of L-Carnitine, Acetyl-L-Carnitine, and Propionyl-L-Carnitine on Body Mass in Type 2 Diabetes Mellitus Patients

**DOI:** 10.3389/fnut.2021.748075

**Published:** 2021-11-08

**Authors:** Dong-Dong Wang, Tian-Yun Wang, Yang Yang, Su-Mei He, You-Mei Wang

**Affiliations:** ^1^Jiangsu Key Laboratory of New Drug Research and Clinical Pharmacy & School of Pharmacy, Xuzhou Medical University, Xuzhou, China; ^2^Department of Pharmacy, Huaian Hospital of Huaian City, Huaian, China; ^3^Department of Pharmacy, The Affiliated Changzhou Children's Hospital of Nantong University, Changzhou, China; ^4^Department of Pharmacy, The Affiliated Suzhou Science & Technology Town Hospital of Nanjing Medical University, Suzhou, China

**Keywords:** l-carnitine, acetyl-l-carnitine, propionyl-l-carnitine, Body Mass, type 2 diabetes mellitus

## Abstract

**Purpose:** The study aimed to explore the effects of l-carnitine, acetyl-l-carnitine, and propionyl-l-carnitine on Body Mass in type 2 diabetes mellitus (T2DM) patients.

**Methods:** Randomized controlled trial (RCT) studies of l-carnitine, acetyl-l-carnitine, and propionyl-l-carnitine in T2DM patients were searched. The change rates of Body Mass index (BMI) from baseline values were used as an evaluation indicator. The maximal effect (E_max_) model by non-linear mixed-effect modeling (NONMEM) was used as the evaluation method.

**Results:** A total of 10 RCT studies, 1239 T2DM patients were included for analysis, including eight studies of l-carnitine, one study of acetyl-l-carnitine, and one study of propionyl-l-carnitine. The study found that l-carnitine could reduce the Body Mass of T2DM patients. Based on only one study each for acetyl-l-carnitine and propionyl-l-carnitine, no significant effects were found in acetyl-l-carnitine or propionyl-l-carnitine. In addition, in order to achieve a plateau of efficacy (80% E_max_), 2 g/day l-carnitine was required for at least 2 weeks.

**Conclusions:** Two g/day l-carnitine was required for at least 2 weeks to affect Body Mass in T2DM patients, and no significant effects were found in acetyl-l-carnitine or propionyl-l-carnitine.

## Highlights

- The present study analyzed the effects of l-carnitine, acetyl-l-carnitine, and propionyl-l-carnitine on Body Mass in T2DM patients.
- L-carnitine could reduce the Body Mass of T2DM patients, in which 2 g/day l-carnitine was required for at least 2 weeks.
- No significant effects on Body Mass were found from acetyl-l-carnitine or propionyl-l-carnitine in T2DM patients.

## Introduction

Type 2 diabetes mellitus (T2DM), a chronic degenerative disease where the pancreas cannot produce enough insulin and/or the insulin produced is inefficient, causing hyperglycemia, is a major health problem and one of the top 10 causes of mortality worldwide (1). According to the International Diabetes Federation (IDF) (2019), 9.3% of adults around the world, amount to 463 million people, have T2DM (1). This number is expected to increase increase to 700 million people by 2045, which is equivalent to 10.90% of the adult population worldwide (1). In addition, T2DM is also an important risk factor for chronic kidney disease, cardiovascular disease, and mortality (2).

From a clinical point of view, T2DM patients are often accompanied by obesity, atherosclerotic disease, dyslipidemia, and hypertension (3, 4), in which more than 50% of T2DM patients have been reported to be obese (3, 5). Overweight or obesity in T2DM can increase the cardiovascular disease risk and further increase the risk of death, which are important determinants of the prognosis in T2DM patients (5, 6). Therefore, intensive therapy for T2DM patients with overweight or obesity is crucial (2).

At present, many drugs have been used to control blood glucose and Body Mass in T2DM patients, among which Wang et al. report the quantitative efficacy of l-carnitine supplementation on glycemic control in T2DM patients (7). However, the effects of l-carnitine, as well as its other forms of existence, acetyl-l-carnitine, and propionyl-l-carnitine on Body Mass in T2DM patients are still unclear. The present study is to explore the effects of l-carnitine, acetyl-l-carnitine, and propionyl-l-carnitine on Body Mass in T2DM patients.

## Methods

### Literature Search and Data Extraction

We searched and extracted the Pubmed database (https://pubmed.ncbi.nlm.nih.gov/) with the deadline of April 2021. Only English publications were included. The terms “l-carnitine,” “acetyl-l-carnitine,” “propionyl-l-carnitine,” and “type 2 diabetes mellitus” were used in the present search strategy. Inclusion criteria included: (I) randomized controlled trial (RCT), (II) with Body Mass Index (BMI) information, (III) exact dose and duration of l-carnitine, acetyl-l-carnitine, and propionyl-l-carnitine. Source, country, grouping, sample size, age, duration of treatment *et al* were extracted from the above-included studies.

In order to eliminate the potential baseline effect, the efficacy of l-carnitine, acetyl-l-carnitine, and propionyl-l-carnitine were evaluated using BMI change rate from the baseline value. The Formula (1) was as follows:


(1) $$\begin{array}{l} \text{E}\%=\frac{{\text{E}}_{\text{t}}-{\text{E}}_{\text{b}}}{{\text{E}}_{\text{b}}}\times 100\% \end{array}$$

E_t_, the value of BMI at time t; E_b_, the value of BMI at baseline.

### Model Establishment

The E_max_ model was used to evaluate the effects of l-carnitine, acetyl-l-carnitine or propionyl-l-carnitine on Body Mass in T2DM patients. In addition, in order to acquire the actual effects on BMI from l-carnitine, acetyl-l-carnitine, and propionyl-l-carnitine, the control effects need to be subtracted from the sum effects. The Formulas (2) and (3) were as follows:


(2) $$\begin{array}{l} {\text{E}}_{\text{D},\text{i},\text{j}}={\text{E}}_{\text{I},\text{i},\text{j}}-{\text{E}}_{\text{C},\text{i},\text{j}} \end{array}$$

(3) $$\begin{array}{l} {\text{E}}_{\text{D},\text{i},\text{j}}=\frac{{\text{E}}_{\text{max},\text{ i},\text{ j }}\times \text{ Time}}{\text{E}{\text{T}}_{50,\text{ i},\text{ j}}+\text{ Time}}+\frac{{\text{ε}}_{\text{i},\text{ j}}}{\sqrt{\frac{{\text{N}}_{\text{i},\text{ j}}}{100}}} \end{array}$$

E_I,i,j_, the sum effects on BMI from l-carnitine, acetyl-l-carnitine or propionyl-l-carnitine, including actual effects and control effects; E_D,i,j_, the actual effects on BMI; E_C,i,j_, the control effects on BMI; i, different studies; j, the time point of every study; E_max_, the maximal effects on BMI; ET_50_, the treatment duration to reach half of the maximal effects on BMI; ε_i,j_, the residual error of study i with j time; N_i,j_, the sample size in study i with time point j. ε_i,j_ was weighted by sample size, assumed to be normally distributed, with a mean of 0 and variance of σ^2^/(N_i,j_/100).

The inter-study variability was described by exponential error or additive error models. The Formulas (4)–(7) were as follows:


(4) $$\begin{array}{l} {\text{E}}_{\text{max},\text{i},\text{j}}={\text{E}}_{\text{max}}\times \text{exp}\left({\text{η}}_{1,\text{i}}\right) \end{array}$$

(5) $$\begin{array}{l} {\text{ET}}_{50,\text{i},\text{j}}=\text{E}{\text{T}}_{50}\times \text{exp}\left({\text{η}}_{2,\text{i}}\right) \end{array}$$

(6) $$\begin{array}{l} {\text{E}}_{\text{max},\text{i},\text{j}}={\text{E}}_{\text{max}}+\;{\text{η}}_{1,\text{i}} \end{array}$$

(7) $$\begin{array}{l} {\text{ET}}_{50,\text{i},\text{j}}=\text{E}{\text{T}}_{50}+\;{\text{η}}_{2,\text{i}} \end{array}$$

η_1,i_, η_2,i_ were the inter-study variabilities, when available, they would be added into E_max_, and ET_50_, respectively. η_1,i_, η_2,i_ were assumed to be normally distributed, with a mean of 0 and variance of ω_1,i_^2^, ω_2,i_^2^, respectively.

In addition, continuous covariates and categorical covariates were evaluated by Formulas (8)–(9) and (10):


(8) $$\begin{array}{l} P_{\text{p}}=P_{\text{T}}+\left(COV-COV_{\text{m}}\right)\cdot {\text{θ}}_{\text{c}} \end{array}$$

(9) $$\begin{array}{l} P_{\text{p}}=P_{\text{T}}\times \;{\left(COV/COV_{\text{m}}\right)}^{\text{θc}} \end{array}$$

(10) $$\begin{array}{l} P_{\text{p}}=P_{\text{T}}+COV\times \;{\text{θ}}_{\text{c}} \end{array}$$

P_p_, the parameter for a patient with a covariate value of COV; P_T_, the typical value of the parameter; COV, covariate; COV_m_, the median value of covariable in the population. θ_c_, a correction coefficient of the covariate to the model parameter.

The model development was done using non-linear mixed-effect modeling (NONMEM, edition 7, ICON Development Solutions, Ellicott City, MD, USA). When a basic model was built, potential covariates were considered for adding into E_max_. The change of objective function value (OFV) was used as the covariate inclusion criteria. When the decrease of OFV was >3.84 (χ^2^, α = 0.05, d.f. = 1), it was considered sufficient for inclusion. When the increase of OFV was >6.63 (χ^2^, α = 0.01, d.f. = 1), it was considered sufficient for significance in the final model (8).

### Model Validation and Prediction

The goodness-of-fit plots of the model (individual predictions *vs*. observations), distribution of conditional weighted residuals (CWRES) for the model (density vs. CWRES, and quantiles of CWRES *vs*. quantiles of normal), and individual plots from different studies were used to estimate the final model. Prediction-corrected visual predictive check (VPC) plots were used to assess the predictive performance of the final model. In addition, the medians and 2.5th−97.5th percentiles of the results from bootstrap (Simulation, *n* = 1,000) were used to compare with final model parameters. The efficacy prediction of l-carnitine on BMI in T2DM patients was simulated by the Monte Carlo method.

## Results

### Included Studies

**Figure 1:**
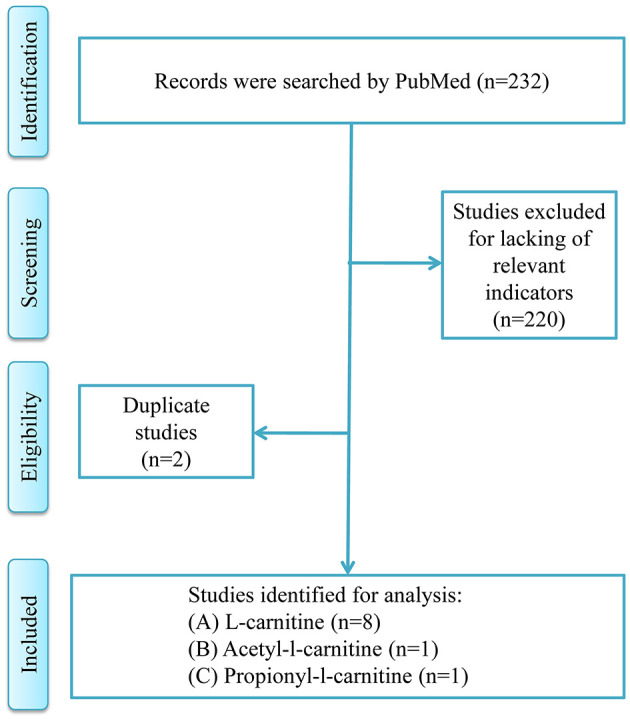
Overview of the strategy for literature review.

**Figure 2:**
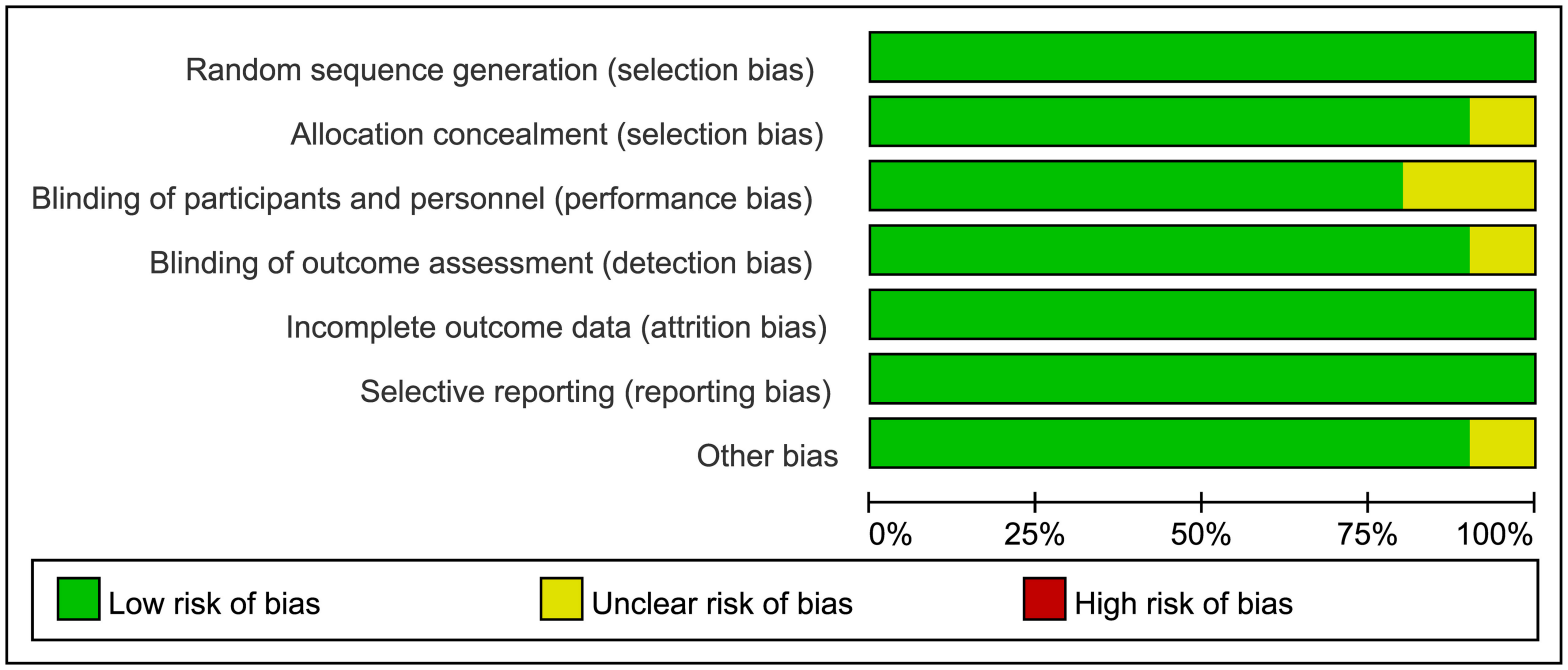
Risk of bias analysis.

Figure 1 was the retrieval process and a total of 10 RCT studies, comprising 1,239 T2DM patients were included for analysis, including 8 studies of l-carnitine (9–16), 1 study of acetyl-l-carnitine (17), and 1 study of propionyl-l-carnitine (18). The dosages of l-carnitine, acetyl-l-carnitine, and propionyl-l-carnitine were 2–3, 2, and 2 g/day, respectively, in the included studies, and the details were shown in Table 1, and part of the literature was retrieved from the previous study (7). The risk of bias analysis was shown in Figure 2. As both acetyl-l-carnitine, and propionyl-l-carnitine had only 1 study, model-based meta-analysis (MBMA) could not be performed at this time for them. Further analysis found that no significant effects on BMI in acetyl-l-carnitine or propionyl-l-carnitine in T2DM patients. Therefore, the following MBMA analysis was mainly aimed at l-carnitine.

**Table 1:** Included randomized controlled studies.

**Study**	**Country**	**Group**				**Sample size**		**Age**		**Duration**
		**Intervention**			**Control**	**Intervention**	**Control**	**Intervention**	**Control**	
El-Sheikh et al. (16)	Egypt	2 g/day L-carnitine	+	4 mg/day glimepiride	4 mg/day glimepiride	31	27	50.9 ± 8.6	50.3 ± 8.8	6 months
Derosa et al. (14)	Italy	2 g/day L-carnitine	+	360 mg/day orlistat	360 mg/day orlistat	132	126	51.0 ± 4.0	53.0 ± 6.0	12 months
Derosa et al. (15)	Italy	2 g/day L-carnitine	+	10 mg/day sibutramine	10 mg/day sibutramine	129	125	54.0 ± 5.0	51.0 ± 4.0	12 months
Malaguarnera et al. (13)	Italy	2 g/day L-carnitine	+	20 mg/day simvastatin	20 mg/day simvastatin	40	40	47.0 ± 13.0	45.0 ± 12.0	12 weeks
Malaguarnera et al. (12)	Italy	2 g/day L-carnitine	+	placebo	placebo	41	40	49.0 ± 13.0	48.0 ± 11.0	3 months
Galvano et al. (11)	Italy	2 g/day L-carnitine	+	20 mg/day simvastatin	20 mg/day simvastatin	38	37	52.1 ± 8.1	51.4 ± 7.6	4 months
Derosa et al. (10)	Italy	2 g/day L-carnitine	+	Placebo	Placebo	46	48	52.0 ± 6.0	50.0 ± 7.0	6 months
Liang et al. (9)	China	3 g/day L-carnitine	+	Placebo	Placebo	23	23	59.4 ± 1.7	57.9 ± 2.6	12 weeks
Parvanova et al. (17)	Italy	2 g/day Acetyl-L- carnitine	+	Placebo	Placebo	109	110	64.9 ± 7.7	64.6 ± 7.5	6 months
Santo et al. (18)	Italy	2 g/day Propionyl-L-carnitine	+	Placebo	Placebo	37	37	61.75 ± 3.03	61.26 ± 1.6	12 months

*Part of the literature was retrieved from the previous study (7)*.

### Modeling and Validation

The actual drug effects of l-carnitine on BMI in T2DM patients is shown in Table 2, the E_max_ of l-carnitine on BMI in T2DM patients was −1.51% and the ET_50_ of l-carnitine on BMI in T2DM patients was 0.5 weeks. In addition, no covariate (in particular dosage) was incorporated into the E_max_ model, showing there was no significant dose-dependence from l-carnitine efficacy on BMI in T2DM patients in the present study. The E_max_ model of l-carnitine on BMI in T2DM patients was shown in Formulas (11):


(11) $$\begin{array}{l} \text{E}=\frac{-\text{1}.51\%\;\times \text{ Time}}{\text{0}.5\;+\text{ Time}} \end{array}$$

E, efficacy of l-carnitine on BMI; Time, l-carnitine treatment duration.

**Table 2:** Parameter estimates of final model and 95% confidential interval.

**Parameter**	**Estimate**	**Simulation (*n* = 1,000)**		**Bias (%)**
		**Median**	**95% confidence interval**	
E_max_, %	−1.51	−1.51	[−8.82, −0.62]	0
ET_50_, week	0.5	0.5	[0.5, 37.2]	0
ω_Emax_	1.345	1.200	[0.003, 6.982]	−10.781
ω_ET50_	0.003	0.003	[0.003, 9.798]	0
ε	0.414	0.415	[0.159, 0.789]	0.242

*95% confidential interval was showed with 2.5th, 97.5th percentile; E_max_ was the maximal effects; ET_50_ was the treatment duration to reach half of E_max_; ω_Emax_ was the inter-study variability of E_max_; ω_ET50_ was the inter-study variability of ET_50_; ε was the residual error; Bias = (Median-Estimate)/Estimate × 100%*.

**Figure 3:**
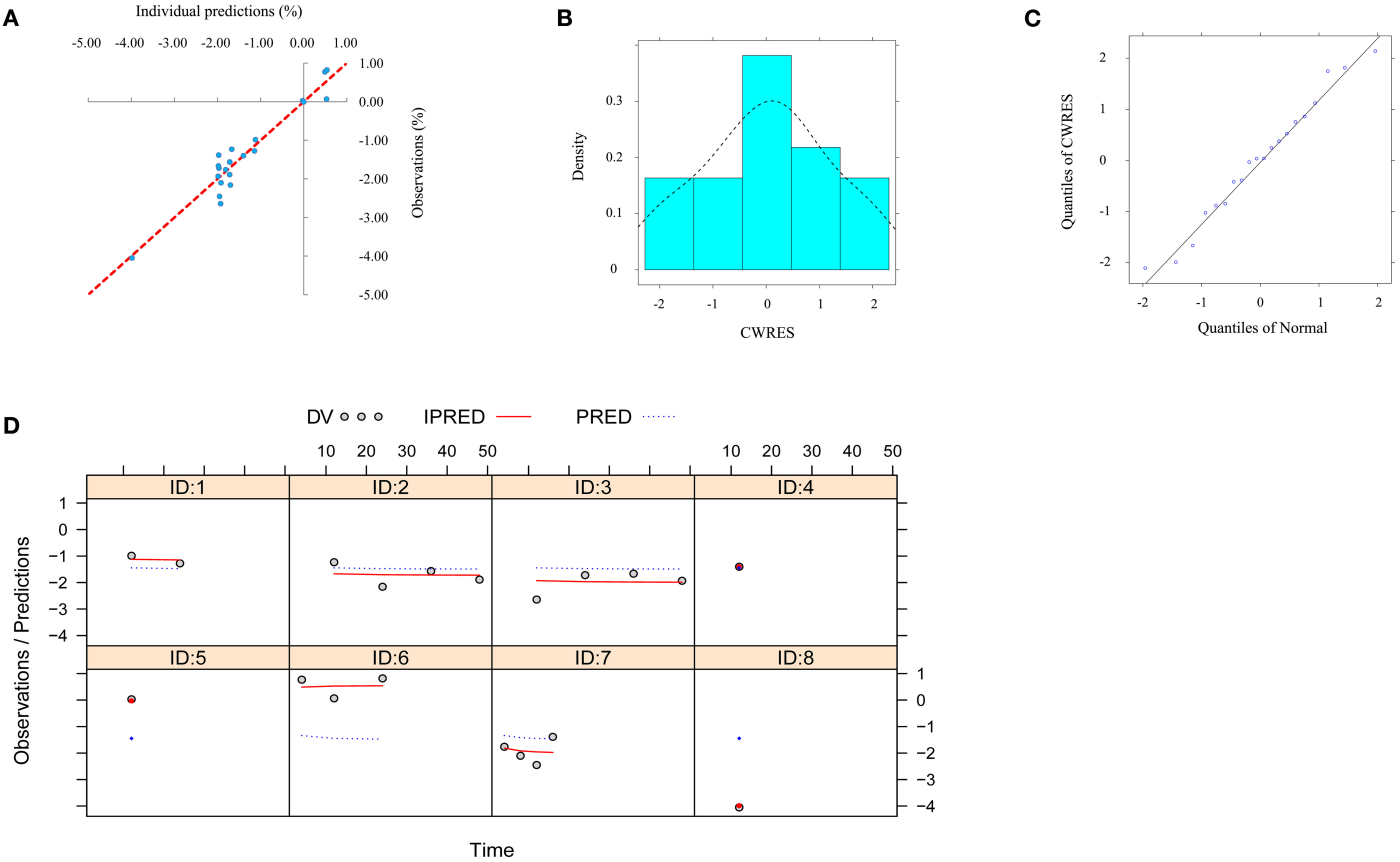
Model evaluation. **(A)** individual predictions *vs*. observations for the model from the effect of l-carnitine on BMI. **(B)** distribution of conditional weighted residuals (CWRES) for model (density *vs*. CWRES). **(C)** distribution of CWRES for model (quantiles of CWRES vs. quantiles of normal). **(D)** individual plots for the model from the effect of l-carnitine on BMI.

The visual inspection of routine diagnostic plots, and individual predictions *vs*. observations, are shown in Figure 3A. The distribution of CWRES for model (density vs. CWRES, and quantilies of CWRES vs. quantiles of normal) are shown in Figures 3B,C. Individual plots from different studies are shown in Figure 3D. As we could see, there were good linear relationships between individual predictions and observations, and individual plots were also consistent meaning the good fitting of the final models. At the same time, the distribution of the model also satisfied the normal distribution.

**Figure 4:**
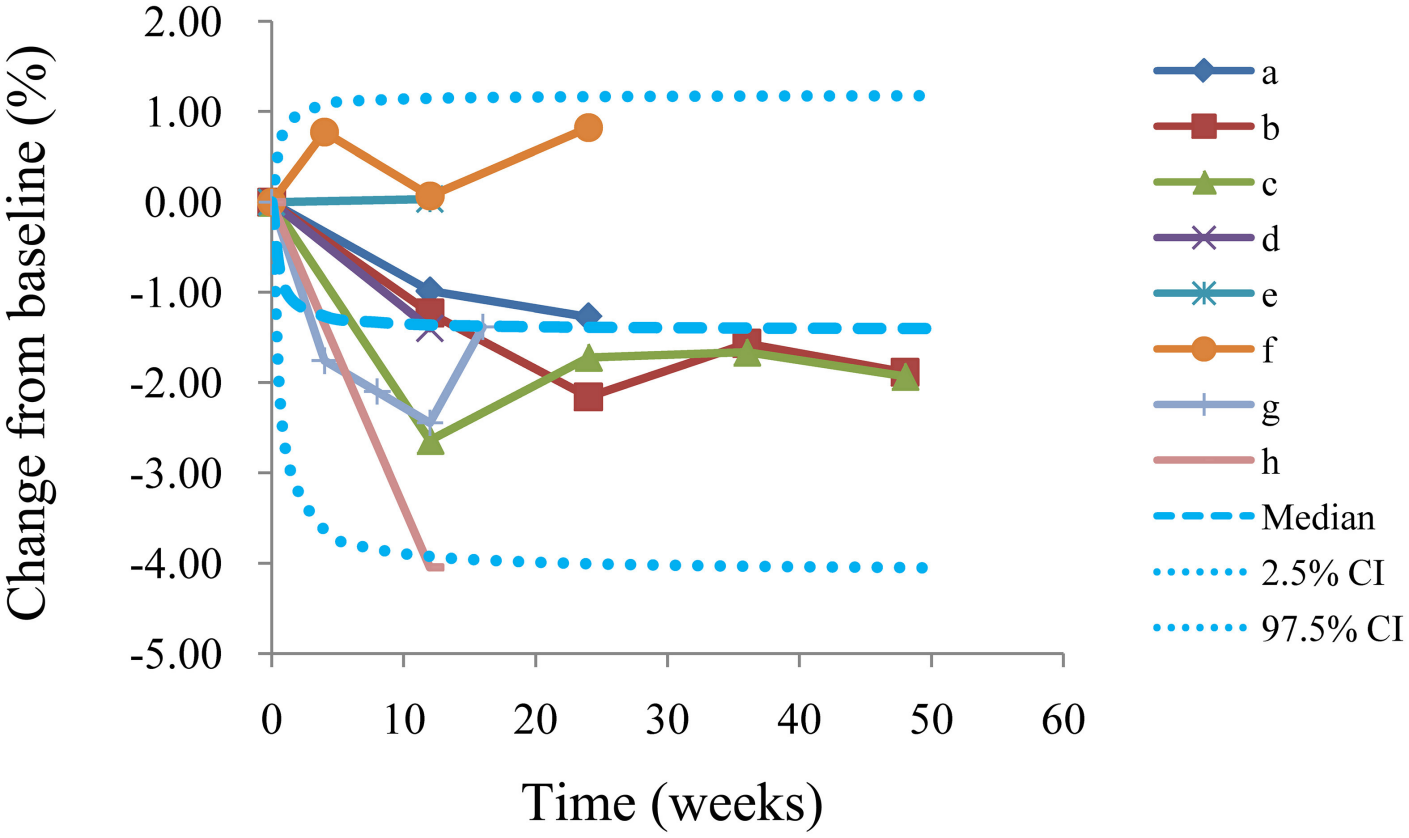
Visual predictive check of the model from the l-carnitine effect on BMI. Median, 2.5% CI and 97.5% CI were simulated by Monte Carlo (*n* = 1,000); CI, confidence interval; From a to h were studies come form El-Sheikh et al. (16), Derosa et al. (14), Derosa et al. (15), Malaguarnera et al. (13), Malaguarnera et al. (12), Galvano et al. (11), Derosa et al. (10), Liang et al. (9), respectively.

The VPC plots are shown in Figure 4, and most observed data were included in the 95% prediction intervals produced by simulation data, which shows the predictive power of the final models.

### Prediction

**Figure 5:**
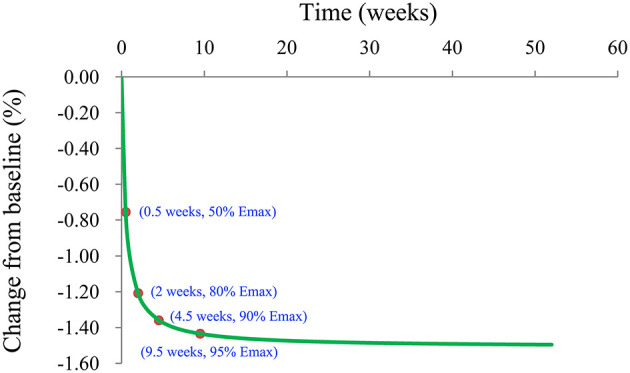
Model prediction.

We also simulated the curve of the final model for the effect of l-carnitine on BMI via the Monte Carlo method. The trend of the efficacy of l-carnitine on BMI in T2DM patients is shown in Figure 5. As we could see from the curve, the efficacy of l-carnitine on BMI at 0.5 weeks was 50% of the E_max_, at 2 weeks was 80% of the E_max_ (plateau stage), at 4.5 weeks was 90% of the E_max_, at 9.5 weeks was 95% of the E_max_. In the current study, the dose range was 2–3 g/day and there was no significant dose-dependence from l-carnitine efficacy on BMI in T2DM patients, so the lower dose of 2 g/day was selected as recommended dose. In addition, in order to achieve a plateau of efficacy (80% E_max_), 2 g/day l-carnitine was required for at least 2 weeks.

## Discussion

Carnitine is derived from amino acids and is found in almost all cells in the body (19). Its name comes from the Latin *carnus*, meaning meat, because the compound is extracted from meat (19). Carnitine is a generic term, which includes l-carnitine, acetyl-l-carnitine, and propionyl-l-carnitine (20). L-carnitine plays an important role in energy metabolism (21). It transfers long-chain fatty acids to cell mitochondria for oxidation, which produces energy needed by the body (21, 22). It also transports harmful substances out of the organelle, preventing them from accumulating in the cell (21). Because of these functions, carnitine is found in high concentrations in skeletal muscle and cardiac muscle cells, which allow them to use fatty acids as an energy source (20). For most people, the body can make enough to meet its needs, but for some people, because of genetic or pharmaceutical reasons, the body cannot produce enough, it is, therefore, an essential nutrient for these individuals (23).

As is well-known, l-carnitine can adjust many events, such as metabolism of glucose and fatty acids, and has the potential to protect these cellular events in several manners including decreasing the production of reactive oxygen species at different points and maintaining mitochondrial functions (24). In addition, it has been reported that l-carnitine had many important pharmacological actions (24–31), for example, l-carnitine has a potential therapeutic effect in treating insulin resistance (32). It is also reported that l-carnitine can improve glycemia in T2DM patients (33). Wang et al.'s report provides valuable quantitative information for the efficacy of l-carnitine supplementation on glycemic control in T2DM patients (7). They find that for the efficacy of l-carnitine on fasting plasma glucose (FPG), 2 g/day l-carnitine is required for at least 36.1 weeks; For the efficacy of l-carnitine on glycated hemoglobin (HbA1c), 2 g/day l-carnitine is required for at least 106 weeks (7). However, the effects of l-carnitine, as well as its other forms of existence, acetyl-l-carnitine, and propionyl-l-carnitine on Body Mass in T2DM patients are still unclear. The purpose of this study is to explore the effects of l-carnitine, acetyl-l-carnitine, and propionyl-l-carnitine on Body Mass in T2DM patients by MBMA.

In the present study, a total of 10 RCT studies comprising 1,239 T2DM patients were included for analysis, including 8 studies of l-carnitine (9–16), 1 study of acetyl-l-carnitine (17), and 1 study of propionyl-l-carnitine (18). The dosages of l-carnitine, acetyl-l-carnitine, and propionyl-l-carnitine were 2–3, 2, and 2 g/day, respectively, in the included studies. Of course, when investigating the efficacy of a drug on Body Mass, important factors should be stable such as diet, antiglycemic drugs, and duration of T2DM. Fortunately, since our study was from RCTs, conditions in the intervention group and the control group were similar in each study. In this way, the control group effects were deducted from the intervention group, and the actual l-carnitine drug effects were obtained. In addition, we also considered the impact of various indicators in different studies on baseline values. In addition, as for both acetyl-l-carnitine, and propionyl-l-carnitine had only 1 study, MBMA analysis could not be performed at this time for them. Further analysis found no significant effects on BMI in acetyl-l-carnitine or propionyl-l-carnitine in T2DM patients.

In further analysis of the effects of l-carnitine on Body Mass in T2DM patients, we found the E_max_ of l-carnitine on BMI in T2DM patients was −1.51% and the ET_50_ of l-carnitine on BMI in T2DM patients was 0.5 weeks. In addition, no covariate (in particular dosage) was incorporated into the E_max_ model, showing there was no significant dose-dependence from l-carnitine efficacy on BMI in T2DM patients. In the current study, the dose range was 2–3 g/day, there was no significant dose-dependence from l-carnitine efficacy on BMI in T2DM patients, so the lower dose of 2 g/day was selected as recommended dose. In addition, in order to achieve a plateau of efficacy (80% E_max_), 2 g/day l-carnitine was required for at least 2 weeks. From the current view, l-carnitine could play an important role in glucose metabolism and increase energy expenditure, meanwhile, l-carnitine had a role in lipid metabolism as well (34–36). For these two reasons, l-carnitine helps Body Mass loss by increasing energy expenditure (36). However, this study had some limitations. The number of studies currently included was limited, and additional studies were needed in the future.

## Conclusions

Two gram per day l-carnitine was required for at least 2 weeks to affect Body Mass in T2DM patients, and no significant effects were found in acetyl-l-carnitine or propionyl-l-carnitine.

## Data Availability Statement

The raw data supporting the conclusions of this article will be made available by the authors, without undue reservation.

## Author Contributions

D-DW, S-MH, and Y-MW conceived and designed the study. D-DW, T-YW, YY, and S-MH collected and analyzed data. D-DW wrote the paper. S-MH reviewed and edited the manuscript. All authors read and approved the final manuscript.

## Funding

This work was supported by the Initializing Fund of Xuzhou Medical University (No. RC20552111), the Fusion Innovation Project of Xuzhou Medical University (No. XYRHCX2021011), the Xuzhou Special fund for promoting scientific and technological innovation (No. KC21257), the Suzhou Science & Technology Town Hospital pre-research fund project (No. 2019Y01), the Suzhou Science and Technology Development Plan Project (No. S YSD2019076), and the Jiangsu Pharmaceutical Society-Tianqing Hospital Pharmaceutical Fund Project (No. Q202024).

## Conflict of Interest

The authors declare that the research was conducted in the absence of any commercial or financial relationships that could be construed as a potential conflict of interest.

## Publisher's Note

All claims expressed in this article are solely those of the authors and do not necessarily represent those of their affiliated organizations, or those of the publisher, the editors and the reviewers. Any product that may be evaluated in this article, or claim that may be made by its manufacturer, is not guaranteed or endorsed by the publisher.
